# Activity of M3814, an Oral DNA-PK Inhibitor, In Combination with Topoisomerase II Inhibitors in Ovarian Cancer Models

**DOI:** 10.1038/s41598-019-54796-6

**Published:** 2019-12-11

**Authors:** Hannah C. Wise, Gopakumar V. Iyer, Kathleen Moore, Sarah M. Temkin, Sarah Gordon, Carol Aghajanian, Rachel N. Grisham

**Affiliations:** 1 Louis V. Gerstner, Jr. Graduate School of Biomedical Sciences, Memorial Sloan Kettering Cancer Center, New York, NY USA; 2 Gynecologic Medical Oncology Service, Department of Medicine, Memorial Sloan Kettering Cancer Center, New York, NY USA; 3 Stephenson Cancer Center, University of Oklahoma, Oklahoma City, OK USA; 4 Hematology/Oncology, Virginia Commonwealth University, Richmond, VA USA; 5 Weill Cornell Medical College, New York, NY USA

**Keywords:** Ovarian cancer, Ovarian cancer

## Abstract

DNA-dependent protein kinase (DNA-PK) has been shown to play a crucial role in repair of DNA double-strand breaks, facilitating nonhomologous end-joining. DNA-PK inhibitors have the potential to block DNA repair and therefore enhance DNA-damaging agents. M3814 is a DNA-PK inhibitor that has shown preclinical activity in combination with DNA-damaging agents, including radiotherapy and topoisomerase II inhibitors. Here we evaluated the activity of M3814 in combination with multiple topoisomerase II inhibitors, doxorubicin, etoposide, and pegylated liposomal doxorubicin (PLD) *in vivo*, utilizing ovarian cancer xenografts. Using cell lines representative of P53 wild-type ovarian cancer (A2780), and P53 mutant ovarian cancer (SKOV3), cells were implanted in the flank of athymic nude female mice. Mice were treated with vehicle, M3814 alone, topoisomerase II inhibitor alone, and M3814 in combination with topoisomerase II inhibitor, and change in tumor volume over time was documented. The addition of M3814 was well tolerated. We demonstrated that M3814 shows limited efficacy as a single agent in ovarian cancer models. The combination of M3814 with PLD showed enhanced activity over PLD as a single agent. Further study of this combination is warranted.

## Introduction

DNA topoisomerases are necessary for normal cell cycle function and survival, and as such, have been successful targets of anticancer drugs. Topoisomerase II (Top2) inhibitors, such as etoposide and anthracyclines, have demonstrated activity against several tumor types, including breast, lung, and ovarian cancer.

Etoposide has been a successful anticancer agent, used to treat a variety of malignancies since the 1980s^1–4^. The drug binds Top2 directly^5,6^ and inhibits the ability of Top2 to religate cleaved DNA, leading to stabilization and accumulation of Top2:DNA cleavage complexes^4,7–9^. Accumulation of these cleavage complexes has a wide range of detrimental effects on cell function, including blocking transcription and replication, and rapid generation of DNA double-strand breaks.

Doxorubicin (Adriamycin) is a potent anthracycline that has been widely used for treatment of solid tumors and hematologic malignancies^10^. The drug is an intercalating agent that binds topoisomerase II (Top2), leading to the formation of Top2:DNA covalent complexes^11,12^. While a number of different mechanisms of action have been proposed for doxorubicin independent of Top2^10^, treatment with doxorubicin results in rapid generation of DNA damage thought to be associated with high levels of Top2:DNA complexes^13,14^.

Although doxorubicin is highly effective, the lack of targeting specifically to the tumor can result in a number of side effects. One of the most prominent risks associated with doxorubicin is cardiotoxicity, which limits dosage, length and efficacy of treatment^15^. Pegylated liposomal doxorubicin (PLD) is FDA-approved for ovarian cancer patients with progressive disease following platinum-based chemotherapy. While doxorubicin was associated with poor response rates in recurrent ovarian cancer, PLD is active^16–18^. However, the outlook for platinum-resistant ovarian cancer patients remains poor, with single agent response rates of approximately 10%, and a median overall survival of approximately 12 months. Combination therapy is a viable option to improve outcome, but a number of trials suggest that combining chemotherapy agents in the platinum-resistant setting leads to increased toxicity with little increase in efficacy^19^.

DNA-dependent protein kinase (DNA-PK) has been shown to play a crucial role in repair of DNA double-strand breaks, facilitating nonhomologous end-joining (NHEJ)^20–23^. This includes repair of double-strand breaks resulting from oxidative stress, oncogene induced transcription, or therapeutic treatment of cancer with chemotherapy or radiation^24^. The active DNA-PK complex is composed of a catalytic serine/threonine protein kinase (DNA-PKcs) and two heterodimeric subunits (KU80 and KU70) that bind to the double-strand break to direct the catalytic subunit to the site requiring repair^24^. The discovery of this necessary role in DNA damage repair highlighted the potential of DNA-PK inhibitors to block DNA repair and therefore enhance DNA-damaging agents. M3814 is a highly potent and selective inhibitor of DNA-PK^25^. M3814 has shown preclinical activity in combination with DNA-damaging agents, including radiotherapy across multiple cancer models^26^. M3814 has also shown activity in combination with the topoisomerase II inhibitor, etoposide, across multiple cell lines derived from lung cancers as well as increased efficacy when added to etoposide and cisplatin in a small cell lung cancer xenograft model, vs etoposide and cisplatin alone^26,27^. In addition, elevated DNA-PKcs expression has been associated with poor cancer specific survival in ovarian cancer patients^28^.

We aimed to determine if DNA-PK inhibitors could enhance the efficacy of type II topoisomerase inhibitors in models of ovarian cancer. We hypothesized that the combination of M3814 and Top2 inhibitors will provide ovarian cancer patients with new treatment options that are both efficacious and tolerable.

## Results

### Ovarian cancer cell lines show etoposide sensitivity *in vitro*

**Figure 1:**
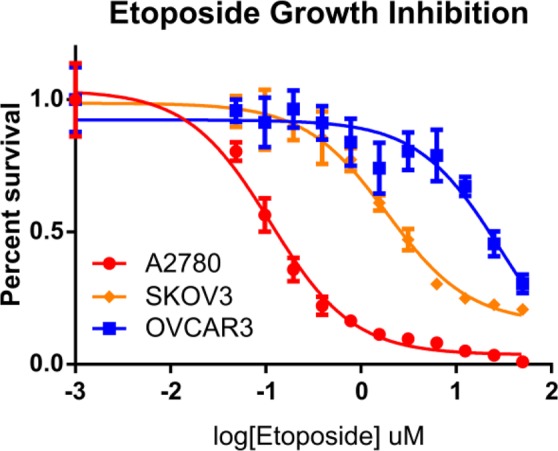
Ovarian cancer cell lines show varied sensitivity to etoposide inhibition. A2780, SKOV3 and OVCAR3 cells were treated with etoposide at varying concentrations. Growth inhibition was measured by MTT assay after 72 hours. IC50’s of each cell line were statistically significant from each other (P < 0.0001, one-way ANOVA).

Because multiple passaging of cell lines has been associated with loss of etoposide sensitivity^29^, we first sought to confirm sensitivity of ovarian cancer cell lines to etoposide *in vitro*. The effect of etoposide on cell proliferation was determined by MTT assay after 72 hours of drug treatment. A2780 and SKOV3 cell lines demonstrated sensitivity to etoposide inhibition with an IC50 of 112 nM and 1.9 uM, respectively. The OVCAR3 cell line was resistant to etoposide inhibition with an IC50 of 29.1 uM (Fig. 1). The IC50 of each cell line was statistically significant from each other (P < 0.0001).

### DNA-PK inhibitor M3814 enhances activity of select topoisomerase II inhibitors *in vivo*

**Figure 2:**
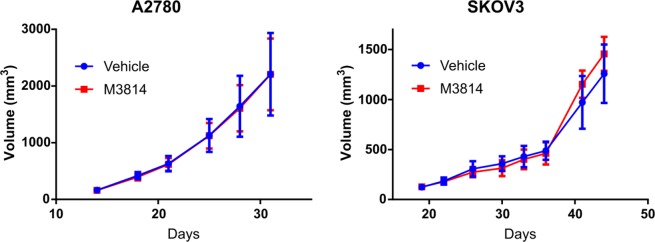
M3814 as a single agent therapy shows limited efficacy. Xenograft experiments were performed with A2780 (left) and SKOV3 (right) cell lines in athymic nude mice to determine efficacy of M3814 as a single agent. Vehicle or M3814 were administered once tumors reached approximately 100 mm^3^ and tumor volume was measured twice weekly.

The DNA-PK inhibitor M3814 has shown activity in combination with etoposide in lung cancer models^27^. To test the efficacy of M3814 in combination with topoisomerase II inhibitors, we performed *in vivo* xenograft studies using cell lines representative of P53 wild-type ovarian cancer (A2780), and P53 mutant ovarian cancer (SKOV3). Cells were implanted in the flank of athymic nude female mice, and drug treatment was started once tumor volume reached approximately 100 mm^3^. Mice were treated with vehicle, M3814 alone, topoisomerase II inhibitor alone, and M3814 in combination with topoisomerase II inhibitor. M3814 treatment alone showed no difference in tumor volume compared to vehicle, in both A2780 and SKOV3 xenograft models (Fig. 2).

**Figure 3:**
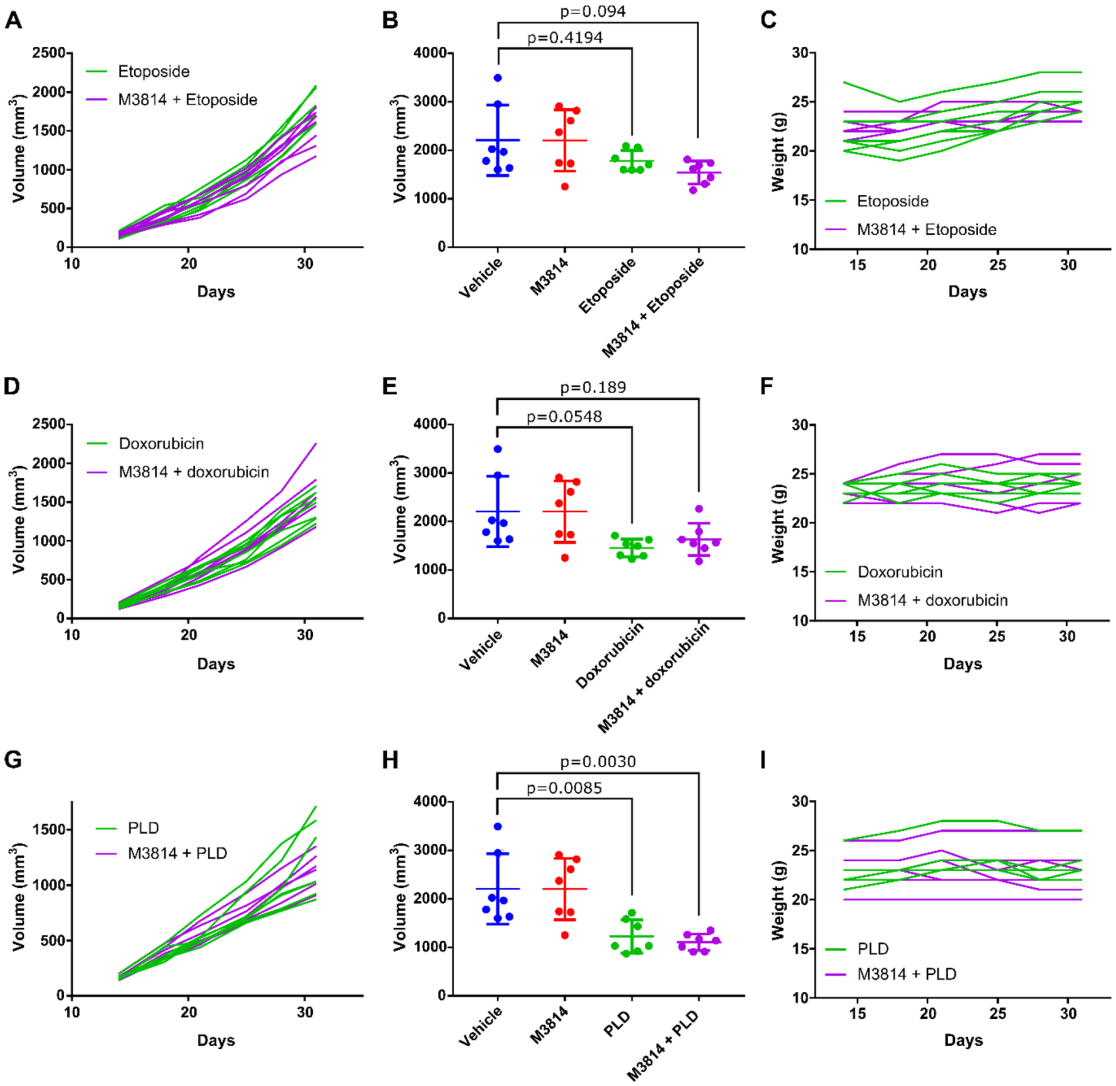
M3814 in combination with DNA-damaging agents in P53 wild-type ovarian cancer cell line model. Xenograft experiments were performed with A2780 cell lines in athymic nude mice. Etoposide (**A**–**C**), doxorubicin (Adriamycin)(**D**–**F**), and PLD (Doxil) (**G**–**I**) were administered alone or in combination with M3814 once tumors reached approximately 100 mm^3^ and tumor volume was measured twice weekly. A, D, and G show tumor volume of individual mice over the course of treatment for single or combination therapy. B, E, and H show average tumor volume at treatment endpoint. One-way ANOVA, n = 7 mice per treatment group. C,F and I show mouse weights during the experiment.

As shown in Fig. 3, A2780 cells demonstrated decreased tumor growth in response to treatment with etoposide, doxorubicin (Adriamycin), and pegylated liposomal doxorubicin (PLD, Doxil) compared to vehicle. Of the single agents, cells were most sensitive to PLD, with a mean tumor volume of 1227 mm^3^ at day 31 compared to a mean tumor volume of 2208 mm^3^ for vehicle alone. Although A2780 cells displayed sensitivity to etoposide *in vitro*, tumor growth *in vivo* did not show inhibition to a similar extent. However, combination of M3814 with etoposide trended toward improved growth inhibition with a mean tumor volume of 1542 mm^3^ at day 31 compared to a mean tumor volume of 1784.1 mm^3^ for etoposide alone, although the difference was not statistically significant (P = 0.8088) (Fig. 3A,B). Similarly, combination of M3814 with PLD also trended toward reduced tumor growth, although not statistically significant, with a mean tumor volume of 1109 mm^3^ at day 31 compared to 1227 mm^3^ for PLD alone (P = 0.9732) (Fig. 3G,H). A2780 showed limited sensitivity to doxorubicin alone *in vivo*, with little improvement seen with combination therapy (Fig. 3D,E). Body weights remained stable throughout the experiment **(**Fig. 3C,F,I**)**.

**Figure 4:**
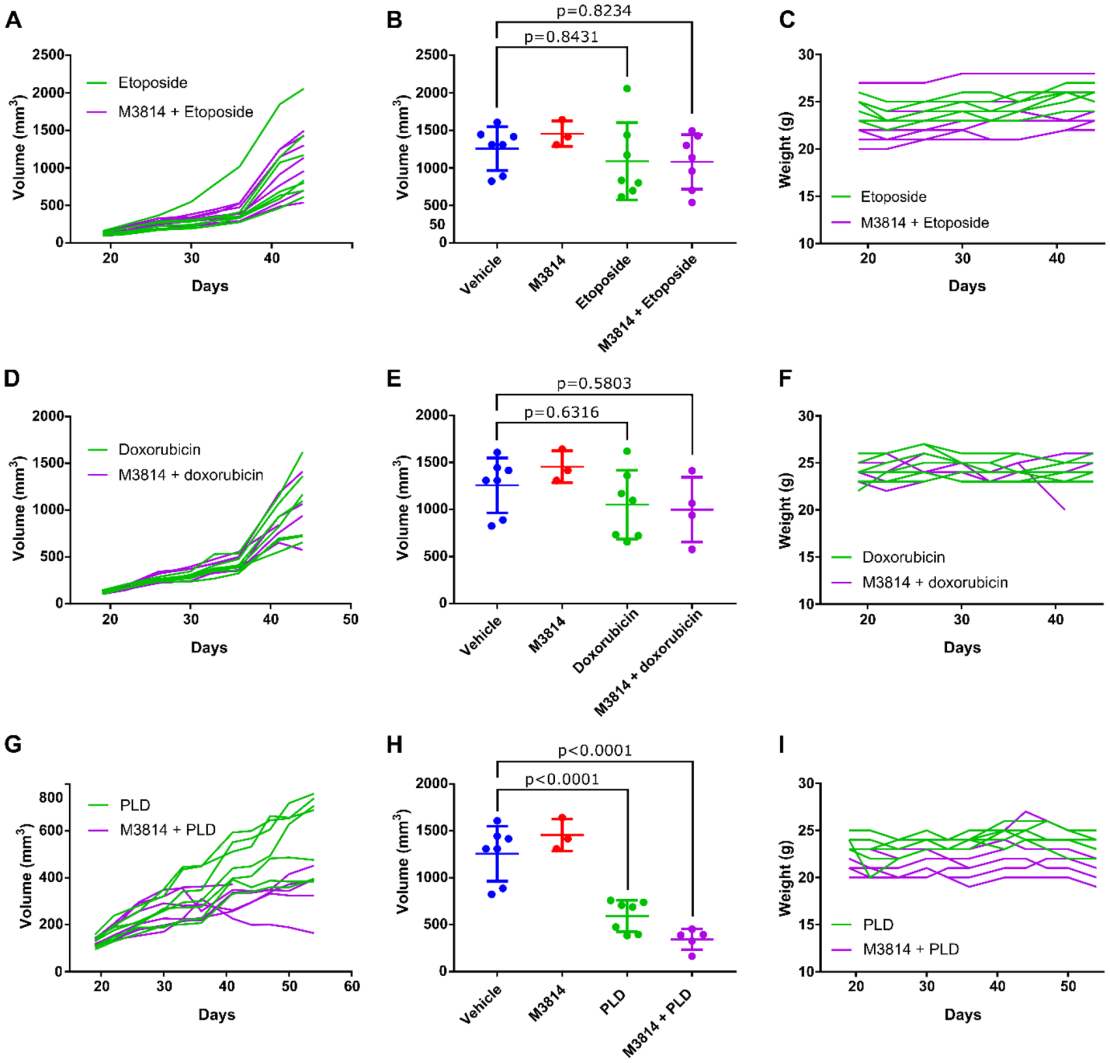
M3814 in combination with DNA-damaging agents in P53 null ovarian cancer cell line model. Xenograft experiments were performed with SKOV3 cell lines in athymic nude mice. Etoposide (**A–C**), doxorubicin (Adriamycin) (**D–F**), and PLD (Doxil) (**G–I**) were administered alone or in combination with M3814 once tumors reached approximately 100 mm^3^ and tumor volume was measured twice weekly. A, D, and G show tumor volume of individual mice over the course of treatment for single or combination therapy. B, E, and H show average tumor volume at treatment endpoint. One-way ANOVA, n = 7 mice per treatment group. C,F and I show mouse weights during the experiment.

Xenograft experiments with the SKOV3 cell line exhibited slightly more resistance to etoposide and doxorubicin compared to the A2780 line, which recapitulated our *in vitro* results. As a result, combination of M3814 with either etoposide or doxorubicin had little effect on SKOV3 tumor growth compared to etoposide or doxorubicin alone (P > 0.9999, P = 0.9934, respectively) (Fig. 4A–E). In contrast, SKOV3 cells were sensitive to PLD, with a mean tumor volume of 593 mm^3^ at day 54 compared to a mean tumor volume of 1257 mm^3^ at day 44 for vehicle. Combination of M3814 with PLD led to a further reduction in tumor growth, with a mean tumor volume of 345 mm^3^ at day 54, although not statistically significant from M3814 alone (P = 0.2143) (Fig. 4G,H). Body weights remained stable throughout the experiment **(**Fig. 4C,F,I**)**.

## Discussion

Treatment options for platinum-resistant ovarian cancer patients remain limited and, although PLD has activity, single agent response rates are low. Viable combination therapy options are necessary to improve the efficacy of available treatment options. DNA-PK inhibitors have been shown activity with DNA-damaging agents, highlighting their potential to improve the efficacy of these agents while remaining tolerable for patients.

We studied the effects of M3814 in combination with topoisomerase II inhibitors. M3814 showed no efficacy as a single agent in ovarian cancer models. This is consistent with the functional mechanism of DNA-PK; inhibiting this protein in the absence of DNA damage should have no effect on the cell. It is only in the presence of DNA damage that DNA-PK inhibition prevents DNA damage repair, exacerbating cell death.

The importance of combining DNA-PK inhibition with therapies that effectively induce DNA damage was emphasized by our *in vitro* and *in vivo* results. In cell lines that demonstrated sensitivity to topoisomerase II inhibitors *in vitro*, combination therapy of M3814 and topoisomerase II inhibitor generally led to reduced tumor growth compared to topoisomerase II inhibitor alone. However, cell lines that were resistant to topoisomerase II inhibitors *in vitro* showed no benefit from combination therapy *in vivo*. The combination of M3814 with PLD produced a more pronounced affect when compared to vehicle in the SKOV3 cell line, which is p53 null, than in the A2780 cell line which is p53 wild-type (p < 0.0001 vs p = 0.003). Prior *in vitro* work of M3814 in combination with irradiation has suggested that p53 mutation may serve as a possible marker for response to M3814^30^. When moving forward with clinical trials for combination therapy, it will be crucial to use DNA-PK inhibitors in combination with DNA-damaging agents that have single agent efficacy, before attempting combination therapy. If the DNA-damaging agent alone does not have an impact on cell growth, then combination therapy with DNA-PK inhibitors will have little success.

In addition to using the right DNA-damaging agents, our results highlight the importance of determining the optimal dosing schedule in therapy to obtain the best result. While the A2780 cell line was sensitive to etoposide inhibition *in vitro*, etoposide had little effect on tumor growth *in vivo*. Because the efficacy of the single DNA-damaging agent alone is critical in effecting any improvement in combination therapy, it will be essential to determine dosing schedules that maximize the efficacy of the single agent.

Although there are important considerations in DNA-PK combination therapy, our results demonstrate that M3814 enhances the efficacy of PLD, leading to reduction in tumor growth. While combination therapy seems necessary to improve upon the available treatment options, recent studies have shown that many combination therapies lead to increased toxicity. Bevacizumab, a humanized monoclonal antibody targeting vascular endothelial growth factor, was recently approved by the FDA for use in combination with chemotherapies (PLD, paclitaxel, and topotecan) in platinum-resistant advanced ovarian cancer, based on a phase III study that demonstrated an improved progression-free survival^31^. However, bevacizumab combination therapy does carry additional risk of toxicity, including risk of hypertension, thromboembolic complications, and bowel perforation, thus precluding its use in certain patient populations^32^. We predict that the DNA-PK inhibitor M3418 may improve the efficacy of PLD in ovarian cancer patients; further clinical trials are warranted.

## Methods

### Cell culture

SKOV3 cells were cultured in DMEM with low glucose, supplemented with 10% Fetal Bovine Serum, penicillin and streptomycin. OVCAR3 and A2780 cells were cultured in RPMI-1640 supplemented with 10 mM HEPES, 10% Fetal Bovine Serum, penicillin, streptomycin, and 0.2 units/mL insulin.

### MTT assay

Cells were plated at 2000 cells per well in a 96-well plate. Outer wells were not used to avoid effects of evaporation. Etoposide was added 24 hours after plating, starting at a concentration of 50 uM and decreasing by 1:2 dilutions, with DMSO as a control. 72 hours after drug was added, cells were incubated with MTT reagent (ATCC®) for 2 hours at 37 degrees, followed by incubation with detergent reagent (ATCC®) for 2 hours at room temperature. Plate was read at 570 nm by microplate spectrophotometer (Epoch™). Absorbance from control media was subtracted out, and readings were normalized to DMSO.

### Xenograft experiments

All animal experiments were performed at Memorial Sloan Kettering’s Research Animal Resource Center and were carried out in accordance with relevant guidelines and regulations. The animal experiments were approved by the Institutional Animal Care and Use Committee (protocol # 04-03-009). For each cell line, two million cells with Matrigel were injected subcutaneously into a single flank of athymic nude female mice. Treatment was started when tumors measured approximately 100 mm^3^. Each treatment group (vehicle, M3814 alone, chemotherapy alone, or combination) consisted of 7 mice. M3814 was administered at 50 mg/kg by oral gavage once daily, five days per week. M3814 vehicle control was 0.5% Methocel™, 0.25% Tween20, 300 mM Na-Citrate Buffer, pH 2.5. Etoposide was administered at 8 mg/kg by intraperitoneal injection once daily, three days per week. Etoposide vehicle was saline. Doxorubicin was administered at 2.5 mg/kg by intraperitoneal injection once weekly. Doxorubicin vehicle was water. PLD was administered at 6 mg/kg by intravenous tail injection once weekly. PLD vehicle was 0.5% dextrose. Tumor growth and animal weight were monitored twice per week. Tumor volume at end of treatment was compared for treatment with vehicle vs chemo and vehicle vs combination therapy for each experiment using an unpaired T-test (Prism) (Figs. 3 and 4).
